# Differences in Unfolded Protein Response Pathway Activation in the Lenses of Three Types of Cataracts

**DOI:** 10.1371/journal.pone.0130705

**Published:** 2015-06-19

**Authors:** Jing Yang, Sheng Zhou, Jianjun Gu, Yujuan Wang, Minfei Guo, Yizhi Liu

**Affiliations:** 1 State Key Laboratory of Ophthalmology, Zhongshan Ophthalmic Center, Sun Yat-sen University, Guangzhou, China; 2 Department of Ophthalmology, The First People’s Hospital of Foshan, Guangdong, China; 3 Department of Ophthalmology, Huichang County People’s Hospital, Jiangxi, China; Medical College of Soochow University, CHINA

## Abstract

**Purpose:**

To investigate the activation of three unfolded protein response (UPR) pathways in the lenses of age-related, high myopia-related and congenital cataracts.

**Methods and Materials:**

Lens specimens were collected from patients during small incision cataract surgery. Lenses from young cadaver eyes were collected as normal controls. Real-time PCR and Western blotting were performed to detect the expression of GRP78, p-eIF2α, spliced XBP1, ATF6, ATF4 and p-IRE1α in the lenses of normal human subjects and patients with age-related, myopia-related or congenital cataracts.

**Results:**

In the lenses of the age-related and high myopia-related cataract groups, the protein levels of ATF6, p-eIF2α and p-IRE1α and the gene expression levels of spliced XBP1, GRP78, ATF6 and ATF4 were greatly increased. Additionally, in the congenital cataract group, the protein levels of p-eIF2α and p-IRE1α and the gene expression levels of spliced XBP1, GRP78 and ATF4 were greatly increased. However, the protein and gene expression levels of ATF6 were not up-regulated in the congenital cataract group compared with the normal control group.

**Conclusions:**

The UPR is activated via different pathways in the lenses of age-related, high myopia-related and congenital cataracts. UPR activation via distinct pathways might play important roles in cataractogenesis mechanisms in different types of cataracts.

## Introduction

Cataract is a clouding of the lens in the eye that affects vision. The process of cataract initiation remains unclear, even though increasing numbers of related mechanisms have been revealed [1–3]. According to several previous reports, cataracts can be induced by a large number of stressors [1], many of which are also endoplasmic reticulum (ER) stressors [4], that induce the accumulation of unfolded protein aggregates in the lens [5–7]. However, multiple intracellular stress pathways ultimately converge at a common event: the unfolded protein response (UPR) [6,8]. The UPR is induced by unfolded protein aggregates in the ER, and the activation of the UPR aims to regulate ER homeostasis upon exposure to environmental changes that cause ER stress [9].

The ER is the site of protein synthesis and protein folding into proper structures [10,11]. Only properly folded proteins are transferred to the Golgi complex for further modification [12]; otherwise, when misfolded proteins accumulate within the ER, the ER chaperone GRP78/BiP dissociates from the UPR sensors PERK, IRE1 and ATF6 and subsequently binds to improperly folded proteins [13,14]. This triggers the activation of these factors and results in the induction of three UPR-related pathways[15]. The IRE1-XBP1 pathway and the ATF6 pathway activation aim to produce a transcriptional response and subsequently increase the capacity of the ER [16]; PERK pathway activation aims to induce temporary translation attenuation [8,17]. These three UPR pathways, which can be activated independently, play distinct roles in regulating various physiological processes [17].

A previous report revealed that many cataracts are caused by unfolded protein aggregates [6]; however, studies on the activation of the three UPR pathways in the lens of different cataract types are rare. Given the important role of the UPR in the initiation and the progression of many types of cataract formation, the present study focused on the differences in UPR pathway activation in the lenses between age-related, high myopia-related and congenital cataracts.

## Materials and Methods

### Patients

The study was approved by the Institutional Review Board/Ethics Committee of Sun Yat-sen University. We certify that all applicable institutional and governmental regulations concerning the ethical use of human volunteers were followed during this study. Thirty Han Chinese patients with age-related cortical cataracts, 30 Han Chinese patients with congenital cataract and 30 Han Chinese patients with high myopia-related cataracts (HM-related cataracts) (−6.0 diopter (D) ≤ SE ≤ -16.0 D) participated in this study; each group was randomly divided into three subgroups for three repeated studies. Nine normal lenses from young cadaver eyes served as the controls in the study. Informed consent was signed by the patients before the study was initiated.

### Human lens specimen collection

Human cataract lens specimens were obtained during small incision cataract surgery at Huichang County People’s and Ganzhou City People’s Hospital in Jiangxi, China. Thirty cortical lens specimens each from Han Chinese patients with age-related cataract, congenital cataract or HM-related cataract were used in the study. The specimens in each group were randomly divided into 3 groups for 3 repeated experiments. The 10 lens specimens in each group were pooled into one sample and then divided into two portions. One portion was used for RNA extraction, and the other portion was used for protein extraction. Nine young and normal cortical lens specimens were obtained from Han Chinese cadaver eyes from the eye bank of Zhongshan Ophthalmic Center, Sun Yat-sen University. The control samples were also randomly divided into 3 groups for 3 repeated experiments, and the specimens in each group were pooled to serve as controls for all other human lens specimens.

### Protein extraction from human lens specimens

Fifty milligram pooled cortical lens specimens were frozen by liquid nitrogen and homogenized for 6mins, the homogenized specimens were lysed in 300μl ice-cold radioimmunoprecipitation assay buffer supplemented with a protease inhibitor cocktail, PMSF, and sodium orthovanadate (Santa Cruz Biotechnology, Santa Cruz, CA) for 5mins, sonicated, and then centrifuged at 13,000× g for 10 min. The supernatants were collected as the protein samples, which were quantified using the BCA protein assay (Thermo Scientific/Pierce BCA Protein Assay Kit) and were analyzed by Western blotting.

### Western blot analysis

A total of 25 μg of protein samples were used for sodium dodecyl sulfate-polyacrylamide gel electrophoresis (SDS-PAGE). The proteins in the gel were then transferred to polyvinylidene fluoride (PVDF) membranes (Bio-Rad). The membranes were blocked in 5% non-fat milk in Tris-buffered saline containing 0.5% Tween-20 (TBST) solution at room temperature for 1 hour and then incubated in the appropriate primary antibodies (diluted in 5% milk in TBST) at 4°C overnight. After fully washing for approximately 30 minutes with TBST, the membranes were incubated in a secondary antibody (diluted in 5% milk in TBST) at room temperature for 90 min. The primary antibody information is as follows: anti-GRP78 (Abcam, Cambridge, MA 1:2000), anti-phospho-IRE1α (Cell signaling, MA 1:1000) and anti-phospho-EIF2α (Cell signaling, MA 1:1000), and anti-ATF6 (Abcam, Cambridge, MA 1:1000). Peroxidase-based detection was performed using chemiluminescence reagent (NEN Life Science). The same membrane was stripped and re-probed using an anti-β-actin antibody (Abcam 1:10000) as the loading control. Densitometric analyses of the Western blots were performed using ImageJ software. We repeated each experiment three times.

### Total RNA extraction from human lens specimens and quantitative real-time PCR

Total RNA was extracted from the human cortical lens specimens using TRIzol (Invitrogen, Carlsbad, CA). For each group, 10 cortical lens specimens were pooled into one biological sample. According to manufacturer’s protocol, approximate 100mg pooled specimen was lysised in 1 ml Trizol, and the experiments were performed in triplicate. Total cDNA was reverse-transcribed using random hexamers and the SuperScript III First Strand Synthesis System (Invitrogen, Carlsbad, CA). According to the Bio-Rad protocols for sample preparation and PCR conditions, we first combined 10 μl of SYBR Green master mix (Bio-Rad) with 3μl of cDNA (1:3 dilution of the first–strand synthesis reaction products) and different primer pairs (1.0 μl of each primer at 1.5 xM each, including primers for GRP78, spliced-XBP1, ATF6, ATF4, and 18S) for a total volume of 20 μl. The reactions were heated for 2 minutes at 50°C-55°C based on the different primers and then for 10 minutes at 95°C, followed by 40 cycles. For each detected gene, the samples were run in triplicate. 18s was used as housekeeping gene. The median of the three cycle threshold (CT) values for each sample was normalized to the CT of 18S rRNA. The data were analyzed via the comparative CT method[18]. The gene-specific primers for Real time PCR were designed with Primer3 software, The gene-specific primer sequences are supplied in Table 1.

**Table 1:** Gene-specific primer sequences for real-time PCR.

Primer	Forward	Reverse
GRP78	5′- GACGGGCAAAGATGTCAGGA-3′	5′- GCCCGTTTGGCCTTTTCTAC-3′
s-XBP1	5′-ACACGCTTGGGAATGGACAC-3′	5′-CCATGGGAAGATGTTCTGGG-3′
ATF6	5'- CTTTTAGCCCGGGACTCTTT-3'	5'- TCAGCAAAGAGAGCAGAATCC-3'
ATF4	5'- GGGACAGATTGGATGTTGGAGA-3'	5'- ACCCAACAGGGCATCCAAGT-3'
18S	5′-TCGGCTACCACATCCAAGGAAGGCAGC-3′	5′-TTGCTGGAATTACCGCGGCTGCTGGCA-3′

### Statistics

All values were expressed as the means ± SD. Statistical significance of the differences in the mean values was assessed using non-parametric ANOVA for the three independent repeats. P-values less than 0.05 were considered to be statistically significant.

## Results

### Up-regulated expression of the ER chaperone GRP78 at the mRNA and protein levels in the different cataract lens groups

**Fig 1:**
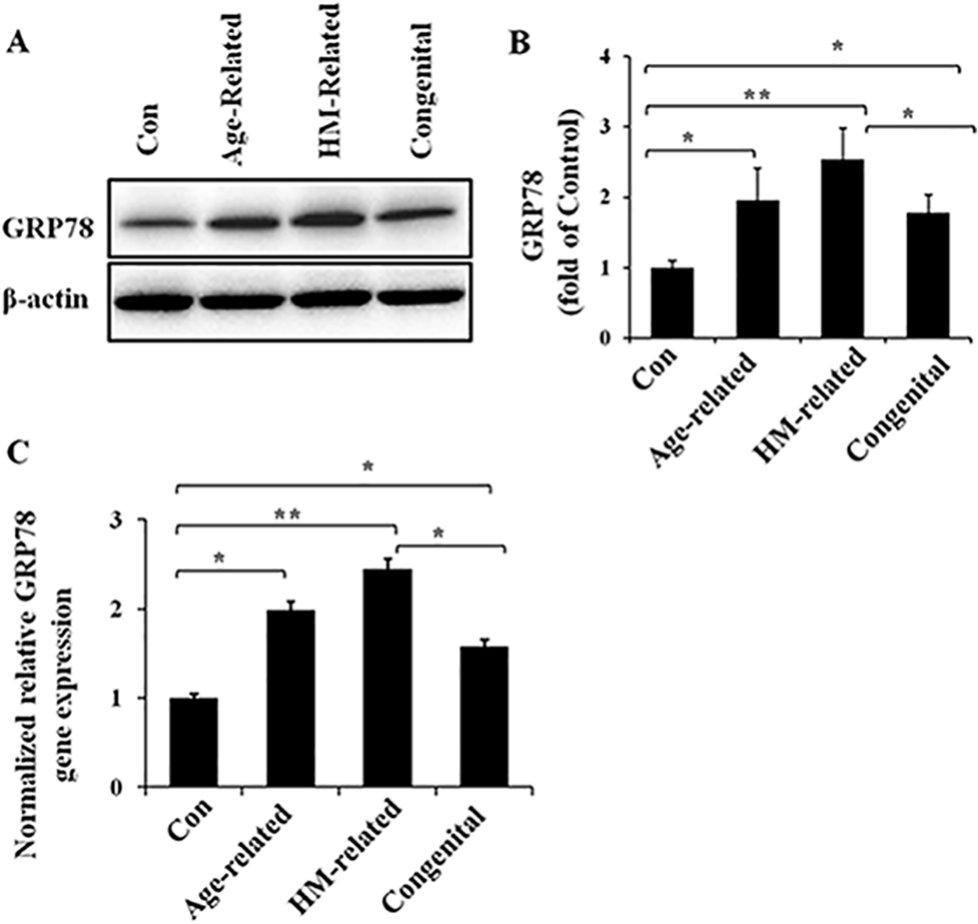
Up-regulated expression of the ER chaperone GRP78 at the mRNA and protein levels in the different cataract lens groups RNA and protein were extracted from human lens specimens. Western blotting was performed to detect the protein expression of GRP78 (Fig 1A and 1B), and β-actin was used as the internal control; real-time PCR was performed to detect the gene expression level of GRP78 in each group (Fig 1C), and 18S rRNA was used as the internal control gene (mean ± SD, *n* = 3). *P < 0.05; **P < 0.001.

To verify the activation of the UPR in the lens of patients with age-related, HM-related and congenital cataracts, we first detected the expression of the ER chaperone GRP78 (BiP) at the protein (Fig 1A and 1B) and mRNA levels (Fig 1C). When we compared the GRP78 protein expression in the lens of these three types of cataracts with the normal control, the GRP78 protein level in the lens of age-related cataracts, HM-related cataracts and congenital cataracts were greatly up-regulated, and this up-regulation was most significant in the lens of HM-related cataracts. Moreover, the mRNA level of GRP78 exhibited similar differences in the lenses of these three different types of cataract. These results showed that GRP78 expression was up-regulated in the lenses of all three cataract groups. However, the up-regulation of GRP78 was associated with the cataract type; the HM-related cataract group exhibited significantly higher up-regulation than the congenital cataract group.

### Activation of the IRE1/XBP1 pathway in the cataract groups

**Fig 2:**
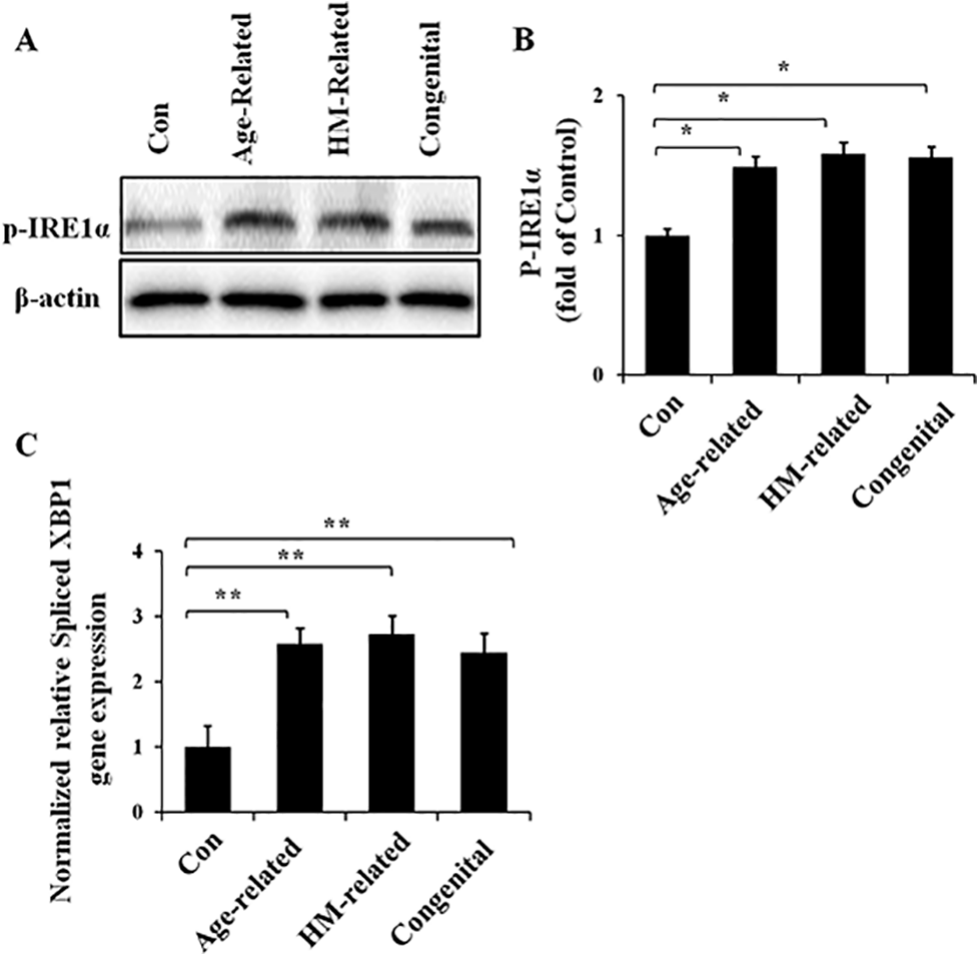
Up-regulated p-IRE1α protein expression level and spliced XBP1 gene expression level in the different cataract lens groups RNA and protein were extracted from human lens specimens. Western blotting was performed to detect the protein expression of p-IRE1α (Fig 2A, Fig 2B), and β-actin was used as the internal control; real-time PCR was performed to detect the relative gene expression levels of spliced XBP1 in each group (Fig 2C), and 18S rRNA was used as the internal control gene (mean ± SD, *n* = 3). *P < 0.05; **P < 0.001.

To investigate the activation of the IRE1/XBP1 UPR pathway, we detected the protein level of phosphorylated IRE1α (p-IRE1α) (Fig 2A and 2B) and the gene expression level of spliced XBP1 (Fig 2C) using Western blot analysis and a quantitative real-time PCR assay, respectively. Protein and RNA samples were extracted from human lens specimens. In our study, the p-IRE1α protein levels were significantly up-regulated in the lens of all three types of cataracts, and no significant differences were detected between the cataract groups. The mRNA levels of spliced XBP1 showed similar trends of up-regulation, with no significant differences between the three cataract groups. These results suggest that the IRE1/XBP1 pathway is activated in the lens of age-related, HM-related and congenital cataract patients.

### Activation of the PERK/eIF2α/ATF4 pathway in the different cataract groups

**Fig 3:**
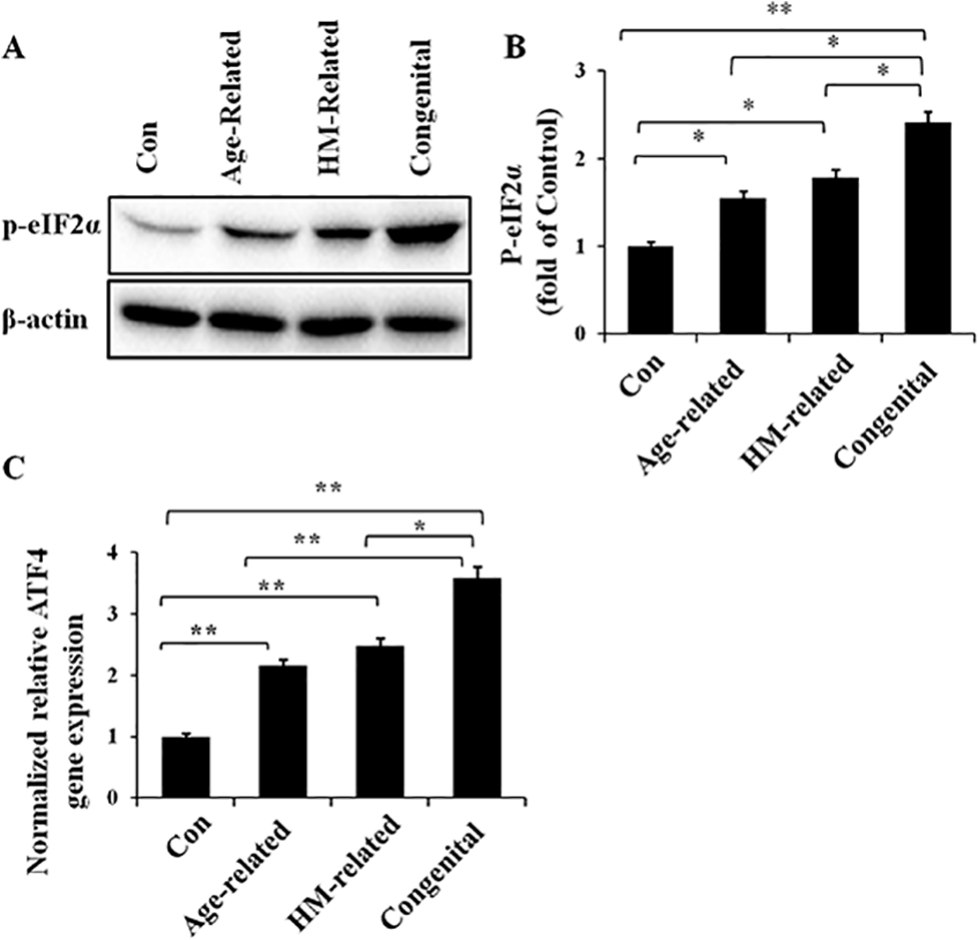
Up-regulated p-eIF2α protein expression level and ATF4 gene expression level in the different cataract lens groups. RNA and protein were extracted from human lens specimens. Western blotting was performed to detect the protein expression of p-eIF2α (Fig 3A and 3B), and β-actin was used as the internal control; real-time PCR was performed to detect the relative gene expression level of ATF4 in each group (Fig 3C), 18S rRNA was used as the internal control gene (mean ± SD, *n* = 3). *P < 0.05; **P < 0.001.

To verify the activation of the PERK/eIF2α/ATF4 pathway in the lens of the three cataract groups, the protein levels of phosphorylated eIF2α (p-eIF2α) (Fig 3A and 3B) and the gene expression of ATF4 (Fig 3C) were detected by Western blot analysis and a quantitative real-time PCR assay, respectively. The protein and RNA samples were extracted from human lens specimens. We found that p-eIF2α protein expression was significantly increased in the lens of all three cataract groups (Fig 3B). The mRNA level of ATF4 showed similar differences between the three cataract groups (Fig 3C). More interestingly, we also found that the protein expression of p-eIF2α and the mRNA level of ATF4 were associated with cataract types; the p-eIF2α protein expression and ATF4 transcription levels in the lens of congenital cataracts were significantly increased compared to the lens of age-related and HM-related cataracts. These findings indicate the activation of the PERK/eIF2α/ATF4 pathway in the lens of age-related, HM-related and congenital cataracts. In addition, the extent of activation of this pathway might be associated with the cataract type.

### Activation of the ATF6 pathway in the cataract lens groups

**Fig 4:**
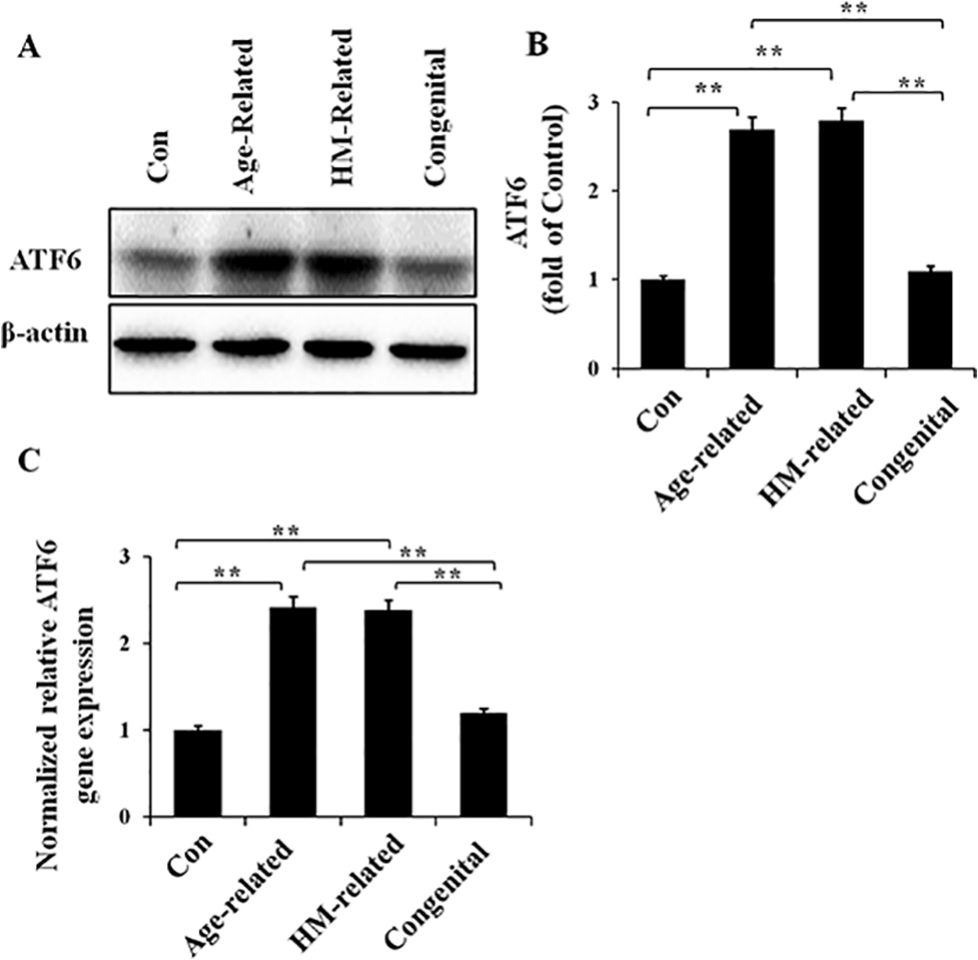
Up-regulated cleaved ATF6 protein and gene expression levels in the different cataract lens groups. RNA and protein were extracted from human lens specimens. Western blotting was performed to detect the protein expression of cleaved ATF6 (Fig 4A and 4B), and β-actin was used as the internal control; real-time PCR was performed to detect the relative gene expression level of ATF6 in each group (Fig 4C), and 18S rRNA was used as the internal control gene (mean ± SD, *n* = 3). *P < 0.05; **P < 0.001.

Finally, we sought to determine whether the ATF6 pathway was also activated in the lens of these three types of cataracts. Western blotting and quantitative real-time PCR were performed on the protein and RNA samples, respectively, extracted from human lens specimens (Fig 4). Compared with the normal controls, the ATF6 protein (Fig 4A and 4B) and mRNA levels (Fig 4C) in the lens of age-related and HM-related cataracts showed similar trends of substantial up-regulation. However, the ATF6 expression level in the lens of congenital cataract did not exhibit significant up-regulation at the protein or mRNA level. These results suggest ATF6 pathway activation in the lens of age-related and HM-related cataracts but not congenital cataracts.

## Discussion

We present here differences in UPR pathway activation in the lens of age-related, high myopia-related and congenital cataracts. ER stress, which can occur under various physiological settings, has significant implications in health and disease [8,19]. Glucose-regulated protein 78 (GRP78), also referred to as BiP, is a central regulator of ER stress due to its role as a major ER chaperone and its ability to control the activation of ER stress sensors (IRE1, PERK, and ATF6) [20]. A large volume of studies established that the induction of GRP78 is a marker of ER stress and is required for alleviating ER stress and facilitating protein folding [21,22]. This is consistent with our findings that GRP78 is significantly induced in the lens of these three types of cataracts (Fig 1). This result also indicates that the lens of age-related, HM-related and congenital cataracts develop ER stress and undergo the UPR.

However, UPR activation includes three branches that can be initiated independently, although there is some crosstalk among them. We first checked the conserved core of the UPR, the IRE1/XBP1 pathway [17]. Our results showed the significant up-regulation of the p-IRE1α protein level and the spliced XBP1 gene expression level. IRE1 includes two subunits, the ubiquitously expressed subunit IRE1α and the tissue-specific subunit IRE1β [23,24], and we chose IRE1α as our target gene. It is well established that when the IRE1α/XBP1 branch is activated, IRE1α autophosphorylates to form p-IRE1α and to splice a 26-nucleotide intron from the mRNA encoding the UPR-specific transcriptional factor XBP1, which results in a frameshift of the XBP1 gene [25]. This frameshift then produces a more stable form of spliced XBP1, which is also a potent activator of UPR genes [26]. Therefore, the increased expression of p-IRE1α-spliced XBP1 in our samples supports the activation of the IRE1/XBP1 pathway in all of these three different types of cataract lenses. In addition, the up-regulated spliced XBP1 can induce downstream genes, including genes encoding ER chaperones such as GRP78, and proteins involved in ER-associated protein degradation (ERAD) [27,28]. This could be another reason for GRP78 up-regulation.

The PERK/eIF2α/ATF4 branch of the UPR is similar to the first pathway described above. Once this branch is activated, PERK undergoes autophosphorylation to form p-PERK, similar to IRE1α. Then, p-PERK phosphorylates the α-subunit of eIF2 to form p-eIF2α [29], which results in global arrest of protein synthesis because of reduced translation [17]. In addition, p-eIF2α can induce another transcription activator, ATF4 [30]. ATF4 then induces a subset of UPR genes [31]. Accordingly, we detected the protein expression of p-eIF2α and the mRNA level of ATF4 in the lens of age-related, HM-related and congenital cataracts, which could represent the activation of the PERK/eIF2α/ATF4 pathway. Significant induction of p-eIF2α and ATF4 was identified in all three types of cataract lenses compared with the normal control lenses. Additionally, the activation of the PERK/eIF2α/ATF4 branch exhibited significant differences between the three types of cataract lenses. The induction of p-eIF2α protein and ATF4 gene expression displayed a more significant change in the lenses of the congenital cataract group than in those of the age-related and HM-related cataract groups.

ATF6, a type 2 transmembrane protein consisting of 670 amino acids, is constitutively expressed as a 90-kDa protein (p90ATF6) [32]. Upon ER stress, ATF6 is cleaved to an N-terminal 50-kDa protein (p50ATF6) [33]. In our study, the induction of cleaved ATF6 protein and ATF6 gene expression in age-related and HM-related cataract lenses suggested ATF6 branch activation in these two types of cataract lenses. However, we did not find any induction of cleaved ATF6 in the congenital cataract lens group, which indicated that the activation of the ATF6 pathway did not occur in the congenital cataract lenses. These results might also indicate a potential reason for the reduced activation of GRP78 in the lenses of the congenital cataract group, because cleaved ATF6 can act as a transcription factor on target genes such as GRP78, GRP94, CHOP and XBP1 [34].

In summary, these results demonstrate the activation of the UPR via distinct pathways in the lens of age-related, high myopia-related and congenital cataracts. All three UPR pathways are activated in age-related and HM-related cataract lenses; however, only the IRE1/XBP1and PERK/eIF2α/ATF4 pathways are activated in the congenital cataract lenses. These findings provide a new explanation for different cataractogenesis mechanisms in different cataract types. Moreover, the prevention of cataracts may entail the removal of any ER stressor in daily life.
